# Editorial: Genetic Architecture and Evolution of Complex Traits and Diseases in Diverse Human Populations

**DOI:** 10.3389/fgene.2022.869056

**Published:** 2022-03-10

**Authors:** Diego Ortega-Del Vecchyo, Jeremy Berg, Mashaal Sohail

**Affiliations:** ^1^ Laboratorio Internacional de Investigación sobre el Genoma Humano (LIIGH), Universidad Nacional Autónoma de México (UNAM), Juriquilla, México; ^2^ Department of Human Genetics, University of Chicago, Chicago, IL, United States; ^3^ Centro de Ciencias Genómicas (CCG), Universidad Nacional Autónoma de México (UNAM), Cuernavaca, México

**Keywords:** evolution, complex traits, complex disease, diversity, genetic architecture

The research topic “*Genetic architecture and evolution of complex traits and diseases in diverse human populations*” presents six review articles and three research studies. Our Research Topic reflects data generation and research performed in Germany, Brazil, United States, Denmark, Estonia, Mexico, United Kingdom and Singapore, spanning countries across the global income spectrum. We present reviews on methodologies to aid complex trait studies in diverse populations, reviews on complex trait studies in locally and globally understudied groups, reviews on studying the evolution and maintenance of genetic variation influencing complex traits using theory and ancient DNA, and new research studies providing resources or insights to understand the genetic architecture of complex traits in Latin America.

With respect to methodologies that aid complex trait studies in diverse populations, Simonin-Wilmer et al. present an overview of strategies for detecting genotype-phenotype associations across diverse populations. With increased interest and movement towards multi-ethnic association studies that promise to improve detection power and prediction accuracy, this review provides a primer for researchers on assessment and control of confounders related to genetic ancestry to help identify true genotype-phenotype associations. Sticca et al. review the recent methodological developments in detecting identity-by-descent (IBD) segments in the genome. These developments enable IBD detection in large biobank-scale datasets and the authors argue for the need to incorporate IBD-based analyses into genetic studies to improve imputation accuracy, the power to detect rare causal variants, and to gain insights into the demographic history of causal variants.

Two articles in our Research Topic reflect on progress conducting complex trait genetics research in groups that have been historically understudied. In his perspective, Charleston Chiang discusses his work on Native Hawaiians as a motivating example to review the parameters of ethical community engagement, along with the challenges and opportunities of conducting genetic studies in minority populations. He reviews how the complex peopling of the Hawaiian islands over several millennia has shaped patterns of genetic variation there, and the hypothesis that the high rates of metabolic disease observed among Native Hawaiians and related Polynesian populations may be linked to ancient genetic adaptations. In their article, Sun et al. review studies of obesity in samples with ancestry from East Asia. Due to several 100 generations of partially independent evolution, genetic studies in east Asian samples are well positioned to discover variants that are at low frequencies or are poorly tagged in European samples. Additionally, inconsistencies between GWAS of obesity in European and east Asian samples point to genetic architectures that only partly overlap, suggesting that the environment may be interacting with genetic effects in different ways in different samples.

Koch and Sunyaev review classical and modern population genetic theory on the maintenance, evolution and distribution of genetic variation for complex traits. There is now strong evidence that trait-associated genetic variation is both highly pleiotropic and shaped by natural selection. While population geneticists have been keenly interested in both of these phenomena for decades, the existing theory is not well suited to the rich but complex data that the field now has access to. Koch and Sunyaev highlight more recently developed theory aimed at bridging this gap, as well as the shortcomings of existing statistical tools and challenges for theoreticians going forward. Further, Irving-Pease et al. discuss what particular insights on the evolution of complex traits can be extracted from the analysis of ancient DNA data. The authors analyze how ancient DNA has been used to study the evolution of traits such as height or skin pigmentation, and also assess the potential to predict phenotypes in archaic hominins. The authors review the prospects of using ancient DNA to detect events of polygenic adaptation, how the degraded ancient DNA data can hinder phenotype studies in ancient individuals and point to strategies to improve complex trait studies using ancient DNA.

This Research Topic also contains three papers that provide new insights on complex traits in Latin American populations. First, Secolin et al. analyze a region on chromosome 8 associated with obesity. A study from Brazilian individuals of the BIPMed cohort, sampled in Campinas, showed that this region is under positive selection and has a high proportion of Native American ancestry compared to the rest of the genome. The authors analyze this region in individuals collected in the cities of Barretos, Ribeirao Preto and Belo Horizonte and find the same overrepresentation of Native American ancestry. Second, Kaibara et al. find that 48 SNPs associated with genetic generalized epilepsies (GGE) in non-Brazilian cohorts do not retain an association with GGE in Brazilian patients using genotype data from 87 Brazilian patients with GGE and 340 Brazilian controls. However, they find that nine SNPs in the imputed flanking 1 Mb region surrounding the 48 SNPs retain an association suggesting that there are some shared variants that impact the risk for GGE in Brazilians and individuals from other cohorts around the world. Finally, Jimenez-Kaufmann et al. show that the inclusion of more genomes from Mexican individuals with a high proportion of Native American ancestry in reference imputation panels improves genotype imputation accuracy for rare variants in admixed Mexican individuals. This result, along with other observations from this study, suggest that improvements in genotype imputation accuracy in Latin American individuals from particular regions will require local sequencing efforts to include more individuals with a high proportion of Native American ancestry in imputation reference panels.

The lofty goal of this research topic was to identify and present state-of-the-art research themes in the genetic architecture and evolution of complex traits and diseases in diverse human populations. The major conceptual and practical research directions and challenges that this Research Topic has helped identify that need to be addressed are 1) Theoretical and conceptual advances to understand trait evolution with clear expectations and assumptions regarding shared and variable genetic architecture across human diversity. 2) The clarification and contextualization of the use of ancestry and populations as sampling and analysis variables in research studies. 3) Methodological advances with transparent assumptions to appropriately analyze complex traits using genetic and environmental data across diverse groups. 4) The construction of local scientific capacity globally to allow for fair and inclusive strategies of data collection, analysis and dissemination of results. 5) Large enough sample sizes across human diversity to allow for meaningful inference. 6) Frameworks incorporating equitable reciprocity for communication with study participants especially those that have been historically marginalized and discriminated against across the globe. In this Research Topic, we present considerations to work towards meeting these challenges. By presenting voices and research done across countries and ethnicities, we show the possibilities of a globally well distributed scientific practice, which we believe is an important precedent for widely representative studies of complex traits and disease.

